# OCT Biomarkers in Neovascular Age-Related Macular Degeneration: A Narrative Review

**DOI:** 10.1155/2021/9994098

**Published:** 2021-07-17

**Authors:** Cristian Metrangolo, Simone Donati, Marco Mazzola, Liviana Fontanel, Walter Messina, Giulia D'alterio, Marisa Rubino, Paolo Radice, Elias Premi, Claudio Azzolini

**Affiliations:** ^1^Ophthalmology Unit, Ospedale di Circolo e Fondazione Macchi, ASST Sette Laghi, Varese, Italy; ^2^Department of Medicine and Surgery, University of Insubria, Varese, Italy; ^3^Multizonal Unit of Ophthalmology of Autonomous Province of Trento, Rovereto, Italy

## Abstract

Age-related macular degeneration (AMD) is the leading cause of legal blindness in elderly people. Neovascular AMD (nAMD) is responsible for the majority of cases of severe visual loss in eyes with AMD. Optical coherence tomography (OCT) is the most widely used technology for the diagnosis and follow-up of nAMD patients, which is widely used to study and guide the clinical approach, as well as to predict and evaluate treatment response. The aim of this review is to describe and analyze various structural OCT-based biomarkers, which have practical value during both initial assessment and treatment follow-up of nAMD patients. While central retinal thickness has been the most common and one of the first OCT identified biomarkers, today, other qualitative and quantitative biomarkers provide novel insight into disease activity and offer superior prognostic value and better guidance for tailored therapeutic management. The key importance of retinal fluid compartmentalization (intraretinal fluid, subretinal fluid, and subretinal pigment epithelium (RPE) fluid) will be discussed firstly. In the second part, the structural alterations of different retinal layers in various stages of the disease (photoreceptors layer integrity, hyperreflective dots, outer retinal tubulations, subretinal hyperreflective material, and retinal pigment epithelial tears) will be analyzed in detail. The last part of the review will focus on how alterations of the vitreoretinal interface (vitreomacular adhesion and traction) and of the choroid (sub-RPE hyperreflective columns, prechoroidal clefts, choroidal caverns, choroidal thickness and choroidal volume, and choroidal vascular index) interact with nAMD progression. OCT technology is evolving very quickly, and new retinal biomarkers are continuously described. This up-to-date review article provides a comprehensive description on how structural OCT-based biomarkers provide a valuable tool to monitor the progression of the disease and the treatment response in nAMD patients. Thus, in this perspective, clinicians will be able to allocate hospital resources in the best possible way and tailor treatment to the individual patient's needs.

## 1. Introduction

Age-related macular degeneration (AMD) is the leading cause of legal blindness in elderly people, especially in developed countries. Its prevalence increases significantly after the age of 50 in each decade, and it affects up to 18% of adults aged over 85 years [1, 2]. In 2020, about 200 million people were affected by AMD worldwide, and the incidence is constantly increasing as a consequence of exponential population aging. The AMD population is expected to be 288 million by 2040 [3, 4].

Neovascular AMD (nAMD) represents a small subset (less than 10%) of total AMD cases; however, the neovascular form is responsible for the majority of cases of severe visual loss in eyes with AMD [5]. It can lead to a progressive and irreversible central visual loss, with severe impairment in daily life. For this reason, appropriate management of this disease is essential.

A multimodal imaging approach should be used in the diagnosis of nAMD, including fluorescein angiography (FA), indocyanine green angiography (ICG), optical coherence tomography (OCT), and OCT angiography. However, OCT can be extremely useful in the follow-up of these patients to predict and evaluate treatment response, as well as to guide treatments [6]. Indeed, it is a widely diffused, user-friendly, quick, and noninvasive device that provides high-resolution in vivo imaging of chorioretinal anatomy and vasculature [7].

Today, with the word “biomarker,” we mean morphological and structural alterations that can provide important information about the stage of a disease [7]. OCT allows to identify specific retinal biomarkers associated with visual acuity (VA) at baseline and to provide information about the patient's visual recovery after anti-Vascular Endothelial Growth Factor (anti-VEGF) treatment, to offer an efficient and personalized management of nAMD.

OCT biomarkers in nAMD can be divided into two different categories. On the one hand, biomarkers based on the retinal distribution of fluids and, on the other, structural biomarkers based on the presence or absence of specific features can be observed in the retinal layers, choroid, or vitreomacular interface. Retinal fluids are observed in exudative AMD and underline the presence of macular neovascularization (MNV), while structural biomarkers can be observed in both exudative and dry AMD as merely manifestations of the progression of the disease.

The measurement of central retinal thickness (CRT) is the most common and one of the first OCT biomarkers identified in the literature [8].

Furthermore, several studies found that fluctuation in retinal thickness due to the activity of the lesion has negative effects on the final VA in patients with nAMD treated with anti-VEGF therapy. Although fluctuation in any retinal tissue compartments has a negative impact on VA, intraretinal cystoid fluid has the worst influence [9]. CRT should be measured in patients with nAMD, because it seems to be correlated with VA both at baseline and after treatment [10], and it is considered the most available and intuitive morphological parameter that can be evaluated from OCT scans. However, studies revealed different results about this correlation, and CRT appears to be not so valuable predictor of visual function. Indeed, studies that consider other parameters such as fluid location, fibrosis, and integrity of individual retinal layers may be more accurate [11]. This is because CRT considers different retinal layers that alone could influence functional outcomes [12, 13]. In particular, intraretinal cystoid fluid has been shown to have the greatest impact on CRT compared with the other fluid features [14]. Intraretinal cysts at baseline may indicate preexisting retinal damage or a more aggressive form of nAMD, reducing visual acuity recovery [15]. For these reasons, we highlight the importance of recognizing the distribution of fluids in retinal layers in nAMD. Specifically, we can distinguish intraretinal cystoid fluid (IRC), subretinal fluid (SRF), and pigment epithelial detachment (PED).

The aim of this narrative review is to describe and analyze various OCT-based biomarkers, which have practical value during both initial assessment and treatment follow-up of patients affected by nAMD.

## 2. Distribution of Retinal Fluids as OCT Biomarker

Neovascular AMD is characterized by the growth of abnormal choroidal vessels, breaking through the Bruch membrane (BM) and proliferating into the subretinal pigment epithelium (RPE) space. These vessels are also defined macular neovascularization (MNV) and can further expand beyond the RPE into the subretinal and intraretinal layers. Exudation due to the immaturity of these vessels often results in fluid accumulation in different layers depending on MNV extension and on retinal tissue ability to solve it [16, 17].

### 2.1. Intraretinal Cystoid Fluid

**Figure 1:**
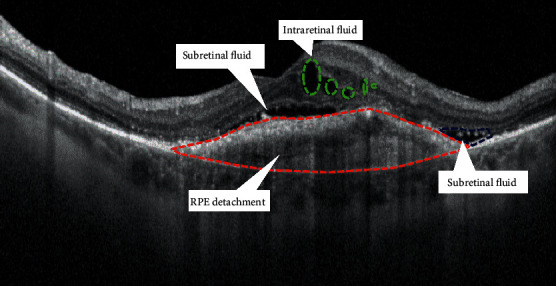
Fluid distribution in neovascular AMD.

Intraretinal cystoid fluid (IRC) can be defined as a cystoid accumulation of fluid within the inner retinal layers, typically associated with increased retinal thickening (Figure 1). IRC is usually related to type 2 and type 3 MNV, but it can also be found in type 1 MNV, in later stages of disease [16]. Many comprehensive literature reviews qualify IRC as the most important negative prognostic biomarker in AMD, associated with a higher risk of visual loss and development of fibrosis or atrophy [7, 18].

IRC presence at baseline is often associated with both poor baseline VA and lower visual improvement after anti-VEGF treatment [6, 19].

In a post hoc analysis of a prospective, randomized multicenter clinical trial including 1240 patients with nAMD treated with intravitreal ranibizumab or aflibercept, Schmidt-Erfurth et al. showed that IRC was the only feature statistically correlated with baseline visual function, with low best-corrected visual acuity (BCVA) values at baseline and during the treatment. When IRC persisted throughout the initial three-month loading phase, there was a further decrease in BCVA, and these degenerative cysts showed the worst prognosis in visual outcomes [20]. Moreover, patients with persistent IRC, despite twelve anti-VEGF injections monthly, showed a higher risk of fibrosis and RPE atrophy [16].

The negative effect of IRC on VA was confirmed in the CATT study at all time points examined in a two-year period. This correlation was stronger in the second year, and the authors postulated that the small hyporeflective cystoid structures persisting after anti-VEGF therapy at the conclusion of the first year may have been due to non-VEGF mechanisms, such as cell death [14].

Finally, we can conclude that IRC is always a negative prognostic biomarker, and, whenever it occurs at baseline or during anti-VEGF therapy, VA would be significantly compromised [20].

### 2.2. Subretinal Fluid

Subretinal fluid (SRF) is described as an exudation occurring between the outer border of photoreceptors and the inner border of the RPE (Figure 1). SRF is the most frequent fluid localization in type 1 MNV, and it can also occur in the context of type 2 MNV. In type 3 MNV, SRF is commonly found in association with IRC overlying PED [16]. The presence of SRF is often associated with higher visual outcomes and lower rates of atrophy, regardless of intravitreal treatment frequency, respect to IRC [16].

A post hoc analysis of prospective, randomized VIEW trials stated that nAMD patients who presented SRF at baseline were shown to have a higher mean BCVA both at baseline and through the duration of anti-VEGF treatment than patients without SRF [19]^.^ A post hoc analysis of prospective, randomized VIEW trials stated that nAMD patients who presented SRF at baseline were shown to have a higher mean BCVA both at baseline and through the duration of anti-VEGF treatment than patients without SRF [19].

Schmidt-Erfurth et al. found that visual prognosis worsened progressively when SRF was associated with retinal Pigmented Epithelium Detachment (SRF + PED) and with IRC (SRF + IRC), with the worst VA in patients with SRF associated with both IRC and PED (SRF + IRC + PED) [20]. Furthermore, refractory SRF may have not a significant negative effect on VA [16, 21], and it was associated with better anatomical and functional outcome than refractory IRC [22].

The FLUID study reported the visual outcomes of nAMD patients treated for 24 months with ranibizumab intravitreal injections in two different treat-and-extend protocols, differing only in the tolerance level of SRF. Patients treated with a protocol that tolerates a small amount of SRF (≤200 *μ*m under the fovea center) achieved a mean BCVA that was noninferior to the group, in which SRF had to be completely resolved [23, 24].

However, the use of microperimetry in the eyes with SRF revealed a progressive decrease in retinal sensitivity over time, expression of functional changes [25].

In the post hoc analysis of the prospective, randomized HARBOR study, baseline SRF absence was associated with an increased risk of macular atrophy (MA) and low VA. The authors provided two different interpretations of these results: SRF itself could be protective against the development of MA; otherwise, SRF may have been related to the presence of a low-activity persistent MNV that limited atrophy, supporting the metabolism of the RPE [18]. However, Sadda et al., disagreeing with the protective role of SRF, emphasized that patients with persistent SRF in the HARBOR study achieved a good visual outcome, because they had been treated continuously during the study [26].

The CATT study showed that eyes with foveal SRF at baseline had higher BCVA at 5 years of follow-up, and this effect was even more evident than at the two-year follow-up. The protective role of SRF was also explained in the CATT study, hypothesizing that SRF might protect retinal photoreceptors from potential toxicity related to direct contact with the underlying diseased RPE. SRF may contain neuroprotective factors providing trophic support to the overlying retina [27].

### 2.3. Sub-RPE Fluid

Serous retinal pigment epithelium detachment (PED) is defined as a separation of the RPE from the inner collagenous layer of Bruch's membrane (Figure 1). Its finding on OCT seems to be less important for the visual prognosis of patients with nAMD than the presence of IRC or SRF.

Previous studies reported inconsistent results regarding the relationship between PED and VA. While some studies associated the presence of PEDs with less favorable visual outcomes, others reported no significant relationships [28].

PED appeared to affect visual recovery only when combined with IRC or SRF, and it is associated with an increase in retreatment frequency [16, 20, 29, 30].

In the post hoc analysis of prospective VIEW trials, 1353 eyes with PED at baseline presented a slightly higher mean BCVA at baseline than patients without PED, but over time, the correlation became less strong and showed a minimal impact of PED on VA [19]. In the post hoc analysis of prospective VIEW trials, 1353 eyes with PED at baseline presented a slightly higher mean BCVA at baseline than patients without PED, but over time, the correlation became less strong and showed a minimal impact of PED on VA [19].

Similarly, in the post hoc analysis of the HARBOR study, patients with PED had better VA at baseline and at month 24 than patients without PED. Indeed, patients who started treatment with higher VA maintained these values until the end of the study. Nevertheless, at year two, there was no statistically significant difference between BCVA increase in patients with PED and patients without PED (*p*=0.08).

A similar rate of MA was seen at month 24 in eyes with and without PED at baseline; however, patients with complete resolution of PED generally developed MA at month 24, regardless of PED size at baseline [31].

In the CATT study, foveal PED was associated with higher VA at year 5. The reason for higher VA in these eyes is unclear, and the authors suggested that sub-RPE fluid provides a trophic support to the retina [27]. Nevertheless, several studies reported a lack of correlation between visual improvement and resolution of PED [31, 32]. Moreover, OCT PED morphology (height, width, volume, dome shape versus peak, presence of RPE tear, or cholesterol bands) was not related to the visual outcome [28].

Finally, PED height reduction was not associated with an increase in VA, suggesting that complete resolution of PED may not influence the final VA, compared to IRC and SRF [33].

## 3. Structural OCT Biomarkers

### 3.1. Retinal Features

#### 3.1.1. Photoreceptor Layer Integrity

**Figure 2:**
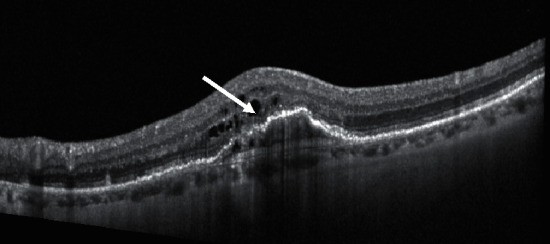
Structural OCT biomarkers: photoreceptors layer degeneration (white arrow).

Photoreceptor degeneration and loss are well-known features in nAMD and are considered key factors of visual decrease in this disease [34] (Figure 2).

Thanks to OCT imaging, it is possible to identify three hyperreflective bands in the photoreceptor layer: External Limiting Membrane (ELM), Ellipsoid Zone (EZ), and Interdigitation Zone (IZ).

Various studies showed that foveal photoreceptor layer integrity is strongly correlated with visual acuity in several retinal diseases [35–40]. In nAMD, the disruption of the foveal ELM band [41–43] and the foveal EZ band [41, 43–45] has been associated with compromised BCVA at baseline and after anti-VEGF therapy.

Restoration of the foveal ELM band and the foveal EZ band after anti-VEGF therapy in nAMD has been described [40, 42, 44]. Restoration of the foveal ELM band after anti-VEGF therapy showed a correlation with better final BCVA. [42].

In a post hoc analysis of the OCTAVE trial, Riedl and colleagues analyzed 185 eyes of 185 newly diagnosed treatment-naïve nAMD patients. They showed a slight positive correlation between foveal EZ integrity and BCVA at baseline [44]. However, within the same study, BCVA variations and modifications in EZ integrity after anti-VEGF treatment did not show a meaningful correlation. Furthermore, Riedl described a correlation between the presence of subretinal fluid and EZ integrity at baseline and the EZ damage with SRF resolution after anti-VEGF treatment.

Coscas and coworkers [42], in a retrospective analysis on 50 eyes with nAMD, described that baseline foveal ELM and EZ integrity showed a predictive value correlating with final photoreceptor layer integrity and final BCVA.

#### 3.1.2. Hyperreflective Dots

**Figure 3:**
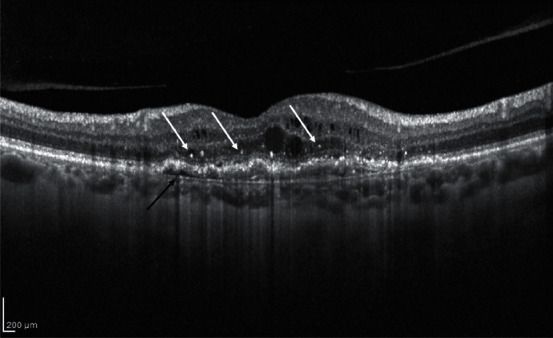
Structural OCT biomarkers: hyperreflective dots (HRD; white arrows) and RPE detachment (black arrow).

Hyperreflective Dots (HRD) are well-defined and circumscribed retinal lesions of approximately 20–40 *μ*m in diameter with equivalent or higher reflectivity than the RPE band on OCT [46] (Figure 3).

HRDs have been described in several retinal diseases such as AMD, diabetic retinopathy, retinal vein occlusion, and central serous chorioretinopathy. In nAMD patients, these lesions are scattered throughout all retinal layers, in particular, around intraretinal cystoid spaces [47].

Since the histopathology of HRDs is unknown, different authors made hypotheses about the structure of this OCT feature.

Curcio and colleagues hypothesized that HRD in nAMD could be composed of two different cell populations: activated migrating RPE cells and lipid-filled microglia cells [48].

Activated migrating RPE cells have been described as discrete hyperreflective lesions in an ex vivo Spectral Domain OCT (SD-OCT) imaging-histology study of two neovascular and two nonneovascular PED. In this study, migrating RPE cells have been found throughout all retinal layers, even surrounding inner retinal capillaries. Furthermore, migrating RPE cells have been found solitarily, as punctate reflective lesions, or in swarms as large irregular lesions [49].

In the same study, a different type of hyperreflective cells was found to be associated with intraretinal cysts in the neovascular PEDs. These cells were larger than RPE, spherical, and full of lipid droplets different from RPE organelles. The authors hypothesized that this cell population could be constituted by microglia [48, 49].

Another study evaluating the association of HRDs with known AMD risk polymorphisms in early forms of AMD showed an association with polymorphisms in genes involved in extracellular matrix interactions, lipid metabolism, and complement activation, suggesting a role of the inflammation in the onset of HRDs [50].

Coscas and colleagues analyzed the prognostic value of HRDs in eyes with neovascular AMD. This study showed that poor BCVA at baseline was significantly associated with persistence of HRDs after anti-VEGF therapy. Moreover, they studied this biomarker after anti-VEGF therapy and showed the persistence of a high number of HRDs in nonresponder patients, while, in responder patients, HRDs quickly decreased after the first injection [51].

#### 3.1.3. Outer Retinal Tubulation

**Figure 4:**
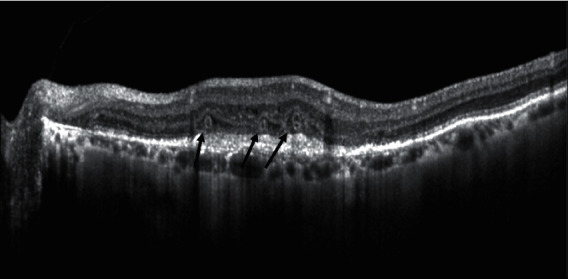
Structural OCT biomarkers: outer retinal tubulations (black arrows).

Outer Retinal Tubulations (ORT) are intraretinal tubular biomarkers located more frequently in the outer nuclear layer, whose OCT aspect was first described by Zweifel et al. in 2009 [52] (Figure 4).

ORTs have been documented in various retinal diseases, including nAMD. The prevalence of ORTs in nAMD is low at the time of first diagnosis but increases over time during anti-VEGF therapy [53, 54].

ORTs are identified as hyporeflective structures surrounded by a hyperreflective band on OCT B scans. Their tubular appearance can be better appreciated in *en face* OCT images.

The hyperreflective outer border of ORTs has been correlated with external limiting membrane and photoreceptor inner segment mitochondria [55].

These lesions can be divided in forming ORTs, without a lumen and with ELM scrolling over a free edge, and formed ORTs. Formed ORTs can be divided in two categories: close ORTs when they have 360° well-defined hyperreflective borders and open ORTs when they show incomplete hyperreflective borders [56].

Preti et al. described a sequential evolution of ORTs; that is, forming ORTs evolve into large open ORTs, which tend to bifurcate into multiple smaller open ORTs. Smaller open ORTs tend to evolve into closed ORTs [57].

Outer retinal tubulations characteristically develop in advanced stages of AMD and are associated with the presence of MNV and geographic atrophy (GA) [56].

Finally, ORTs can be considered as a rearrangement of photoreceptors as a consequence of retinal injury, and their presence has been associated with worse visual prognosis in nAMD patients [53].

Anti-VEGF treatment seems not to decrease the development of newer ORTs neither to enhance the regression of preexisting ORTs [53, 54, 58].

#### 3.1.4. Subretinal Hyperreflective Material

**Figure 5:**
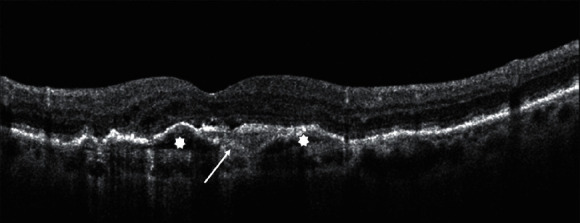
Structural OCT biomarkers: subretinal hyperreflective material (SRHM; white arrow) and pigment epithelium detachment (white stars).

Subretinal hyperreflective material (SHRM) is a tomographic feature seen on OCT as a hyperreflective material located between the neurosensory retina and RPE [59] (Figure 5).

In eyes with nAMD, SHRM is common and often persists after anti-VEGF treatment [60, 61]. The nature of SHRM is still not known, as the literature is lacking direct histopathological studies. Authors suggest that SHRM could be made up of fluid, fibrin, blood, scar, and MNV, even though it could change over time [59, 62].

Willoughby and coworkers, in a post hoc analysis of the CATT study, demonstrated that the presence of SHRM was associated with worse VA in any position, regardless of its size. Furthermore, central fovea position and greater SHRM height and width were correlated with worse BCVA.

Moreover, the persistence of SHRM from baseline to follow-up visits was associated with a lower increase in VA [59].

Pokroy and coworkers, in a retrospective study on 73 eyes treated with three intravitreal bevacizumab injections monthly followed by PRN regimen, showed that the presence of any SHRM within the 1 mm^2^ central fovea predicted a worse visual outcome after twelve months of treatment, especially if well-defined SHRM borders and thicker SHRM were present [63].

Optical coherence tomography angiography (OCTA) can recognize vascular from avascular SHRM components [64]. Dansingani and colleagues identified SHRM subtypes in a cohort of 33 patients. They found that 3 patients showed a type 2 MNV, 4 patients fibrosis or disciform scar, 5 patients macular hemorrhage, 10 patients subretinal hyperreflective exudation, and 17 patients vitelliform lesions [64]. Kawashima and colleagues, in a prospective consecutive interventional case series on 44 treatment-naïve nAMD eyes, showed that vascular SHRM still remains after treatment with three intravitreal aflibercept injections monthly. Based on these findings, the authors suggested that vascular SHRM underlies a lower response to anti-VEGF therapy [65].

Kumar and colleagues, in a retrospective analysis on 499 treatment-naïve nAMD patients enrolled in randomized anti-VEGF and antiplatelet derived growth factor (PDGF) clinical trials, showed that baseline SHRM characteristics, such as layered appearance, hyperreflective spots, SHRM separation from the outer retina, and larger size, had a negative impact on subsequent visual acuity. Furthermore, in the same study, Kumar et al. described that decreasing reflectivity of SHRM lesions at follow-up visits correlated with better visual acuity [66].

#### 3.1.5. Retinal Pigment Epithelial Tears

**Figure 6:**
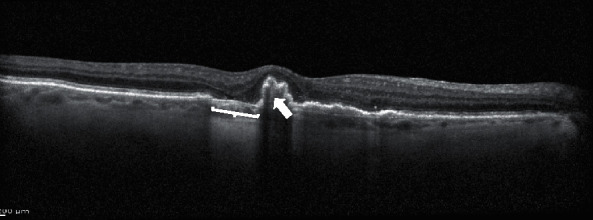
Structural OCT biomarkers: RPE tear (white bracket) and RPE layer retraction (white arrow).

Retinal pigment epithelial (RPE) tears, also known as RPE rips, represent a disruption of the RPE monolayer (Figure 6). RPE tears are a well-known complication of nAMD [67]. In the large majority of cases (86.2%–100%), RPE tears occur in patients with preexisting PEDs [68]. RPE tears could occur spontaneously or as a consequence of thermal laser treatment, photodynamic therapy, or anti-VEGF therapy. The size and recent onset (<4.5 months) of PED are potential risk factors for developing RPE tears [68]. Moreover, different morphologies of PED additionally increase the risk of developing an RPE tear. Indeed, 80.6% of tears come from a fibrovascular PED, 16.2% after a hemorrhagic PED, and 3.2% from serous PEDs [69]. The PED height is a predictive factor according to several authors. Chan et al. reported that PED height greater than 400 *μ*m is the only significant risk factor for an RPE tear after bevacizumab injection, and with PED height over 600 *μ*m, the risk increases [70]. Sarraf et al. described that a height >550 microns is a high-risk factor for the development of RPE tears with ranibizumab therapy [71]. Indeed, this value is considered by a large consensus as the only predictive value for RPE tears occurrence. Chiang et al. noted that, in addition to PED height, a large PED basal diameter on fluorescein angiography was also a risk factor [72]. In addition, a small MNV size/PED size ratio (<50%) has been suggested as a risk factor by Chan et al. [73]. RPE irregularities along the PED borders, such as RPE thinning, RPE indentations, and small interruptions in the PED on OCT, have also been reported as predictors of impending RPE tears in patients with exudative AMD treated with anti-VEGF therapy [74, 75]. Otherwise, Musashi et al. observed microrips at the margin of the PED in 11 patients with polypoidal choroidal vasculopathy treated with photodynamic therapy and noted that no one progressed to a tear. Ten of these microrips disappeared spontaneously [76]. This difference between the studies may be due to different etiologies and treatments [77].

The RPE rip in OCT imaging looks like an area of RPE disruption, usually located between the normal retinal architecture and the border of the PED. Small RPE ruptures appear as a tented up or peaked PED with a microscopic RPE defect. As the grade of the RPE rip increases, OCT shows a wider patch of RPE loss with the redundant RPE taking on a dome-shaped configuration [78]. The retracted RPE is observed with an irregular contour, dense hyperreflectivity from duplication of the RPE, and a shadowing effect beneath it [68, 77]. The overlying neurosensory retina remains intact with or without neurosensory detachment. In the area, where the RPE layer is absent, the bare choroid shows hyperreflectivity and deeper signal penetration [79].

RPE tears must be differentiated from RPE apertures described by Querques et al. in avascular PEDs secondary to AMD. RPE apertures are defined as round discontinuities that can be located not only at the base of an avascular PED, but also at its apex. Importantly, they do not show the rippling or retraction seen in RPE tears. The authors suggested that focal atrophic progression of drusenoid material plays role in their pathogenesis [80].

RPE tear development seems to be due to several factors: firstly, the presence of the PED applies hydrostatic pressure to the RPE and stretches it. The contractile capacity of the MNV adds tangential forces to the RPE monolayer [77]. These processes together create a weak point at the junction of the detached and flat RPE, where the tear occurs. The tear is followed by retraction of the pigment epithelium, revealing bare Bruch's membrane [78]. Several authors propose that intravitreal anti-VEGF treatment could increase the risk of early tearing, possibly by shrinking the neovascular complex, thereby creating an extra contraction force in a PED at risk and triggering the tear of the RPE [2]. Despite multiple reports, not all studies support these conclusions [78]. There is still no evidence that any anti-VEGF agent is safer than the others [78]. In eyes with high-risk, alternative anti-VEGF treatment protocols such as low-dose and frequent injection treatments have been proposed. However, there is no proven method to foresee the development of RPE tears [78].

The visual acuity outcome after an RPE tear is variable and is determined not only by control of neovascularization, but also by tissue remodeling. Poor visual acuity is more frequently observed in cases of foveal involvement [78]. Mukai et al. observed two different repair mechanisms in the area, where RPE tears developed. When the subretinal fluid persists for more than 6 months, the denuded area is covered with thickened proliferative tissue. With an early resolution of the subretinal fluid, the outer retina appeared to be directly attached to Bruch's membrane [81]. Oishi et al. [82] describe that, after an RPE tear, a thinning of the outer nuclear layer occurs in the area devoid of the RPE and in adjacent areas. This means that photoreceptors are lost progressively after the development of an RPE tear.

Once the RPE tear has developed, there are no clinical practice guidelines on the management of these patients. However, the evidence points to continuing (or starting) anti-VEGF treatment, which seems to reduce the development of fibrosis and decreases the risk of disciform scar development. In the presence of an active CNV, anti- VEGF injections should be repeated until the underlying disease has been resolved [68, 77, 83]. Invernizzi et al. suggested that long-term visual outcomes in eyes affected by an RPE tear may be mostly related to the patient's response to therapy than to the tear itself [67].

#### 3.1.6. Vitreomacular Interface Alterations

**Figure 7:**
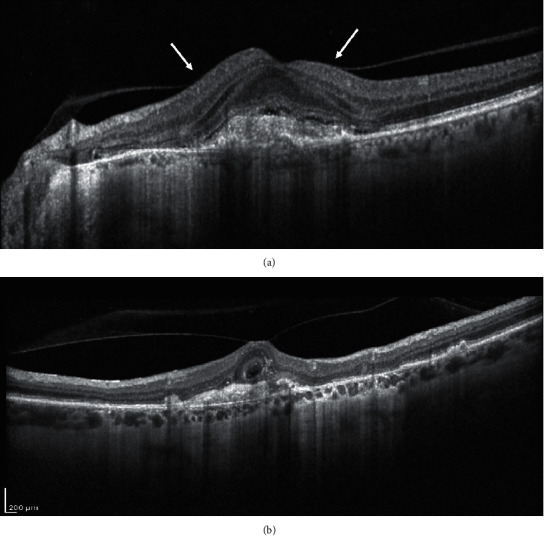
Structural OCT biomarker: (a) vitreomacular adhesion (VMA, white arrows). The posterior hyaloid is partially detached, with a continuous adhesion on the foveal surface; (b) vitreomacular traction. The posterior hyaloid is fully detached but a foveal adhesion is present on the fovea with an evident traction.

The development and progression of AMD has been related to different risk factors. Recently, vitreomacular alterations have been identified as new risk factors for AMD [84]. In particular, thanks to the recent OCT imaging definition of vitreomacular adhesion (VMA) and traction (VMT), many papers describe the role of the vitreous in different aspects of exudative AMD [85]. VMA is characterized by an elevation of the cortical vitreous above the retinal surface, with the posterior hyaloid remaining attached within a 3 mm radius of the fovea without retinal abnormalities; VMT presents perifoveal vitreous cortex detachment from the retinal surface with macular attachment of the posterior hyaloid within a 3 mm radius of the fovea and distortion of the foveal surface [86] (Figures 7(a) and 7(b)).

In recent studies, there was no significant difference in the prevalence of VMA between eyes affected by AMD and age-matched controls [87]. VMA has been evaluated and described in a wide percentage of exudative AMD. Particularly, the adhesion area matched to the site of MNV.

The relation between adhesion and development of exudative MNV has been described not to be causative but correlated: the exudative, fibrotic, and proliferative events make the vitreal adhesion stronger and stabler to the retinal surface [88, 89]. For this reason, the higher prevalence of VMA reported in previous literature might be considered as a consequence of MNV, rather than a causative factor.

While the extension of the neovascular lesion is associated with the presence of VMA, the area of vitreomacular adhesion is not related to the angiographic subtype of the neovascular lesion [90–92]. Even though different opinions describe the role of a preexisting VMA and MNV development [93], the possibility to resolve the VMA may not represent a protective factor in high-risk patients [94].

Several authors describe a higher number of intravitreal injections to treat nAMD in patients presenting VMA compared to patients with complete PVD. The reasons are not fully understood, but VMA seems to make the exudative lesion more extensive and resistant to intravitreal treatment. This fact could be also due to the partial vitreous detachment influencing the achievement of the anti-VEGF therapy target [30, 95–97].

Vitreomacular traction may present a different behavior. As it could be symptomatic by causing alterations to inner and outer retinal layer for its persistent and tractive action on the macular surface, it could lead to the development of a chronic inflammation influencing the progression of exudative AMD, more than VMA [97, 98].

The presence of VMT reduces the functional and morphological improvement at two years in patients treated with anti-VEGF, requiring more injections in a ProReNata (PRN) regimen. Several authors describe the beneficial effects of surgical VMT removal on exudative AMD response to anti-VEGF. It could be due to the reduction of chronic traction and inflammatory effects, as well as to the diffusion of cytokines and VEGF from the macula into the vitreous [99–101].

### 3.2. Choroidal Features

#### 3.2.1. Sub-RPE Hyperreflective Columns

Sub-RPE Hyperreflective Columns are OCT biomarkers that look like narrow columns of hyperreflectivity beneath the RPE. They have been considered as a sign of a weakened or cracked RPE layer, where fluid, blood, and/or vessels can more easily break into the subretinal space. It has been described in 27% of eyes with neovascular AMD. These sub-RPE columns are different from the regions of the increased backscattering effect observable in geographic atrophy, which look like large spans of sub-RPE hyperreflectivity [102, 103].

#### 3.2.2. Prechoroidal Clefts

**Figure 8:**
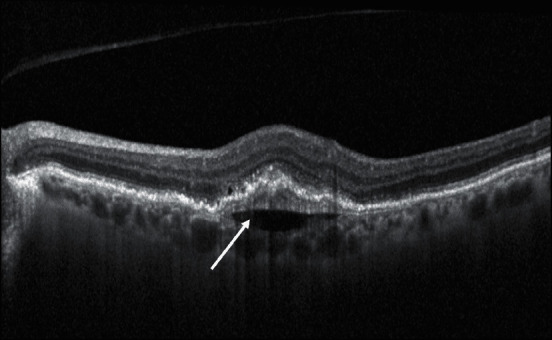
Structural biomarker: prechoroidal clefts (white arrow).

Prechoroidal Clefts are outwardly bowed hyporeflective cavities between the deeper fibrous component and the underlying hyperreflective choroid characterizing the multilayered PED (Figure 8). In eyes with exudative AMD receiving serial intravitreal anti-VEGF injections, chronic fibrovascular PEDs appear to develop through a sequential layering of hyperreflective bands beneath the RPE. Near the base of the PED (adjacent to the choroid), a fusiform complex of homogenous hyperreflective lamella surrounds the main body of this internal structure showing contractile properties, resulting in the spindle-shaped appearance. The progressive modification of sub-RPE neovascular lesions causes a delamination of RPE-Bruch's membrane complex and of the choroidal tissue due to retractive but also exudative forces [104, 105]. A similar lesion has been described by Khan in patients with polypoidal choroidal vasculopathy as one component of a “triple-layer” sign. In the literature, different authors described that these eyes surprisingly maintain a good to excellent visual acuity, probably because the neovascular and cicatricial process is confined to the sub-RPE space and effectively inhibited by continued anti-VEGF therapy. A second hypothesis considered neovascular tissue as a surrogate of the choriocapillaris and provided oxygenation or nutritional support to the outer retinal layers and the RPE, thereby protecting against involution and geographic atrophy [104–108].

The multilayered PED may be at lower risk of developing a high-grade RPE tear due to the stabilizing effect of a fibrovascular tissue complex that fills the sub-RPE space and anchors the PED to the underlying Bruch's complex. The early presentation in the first six months of treatment could have a negative prognostic value, probably due to the associated complications like RPE hemorrhage and RPE rips [106].

#### 3.2.3. Choroidal Caverns

**Figure 9:**
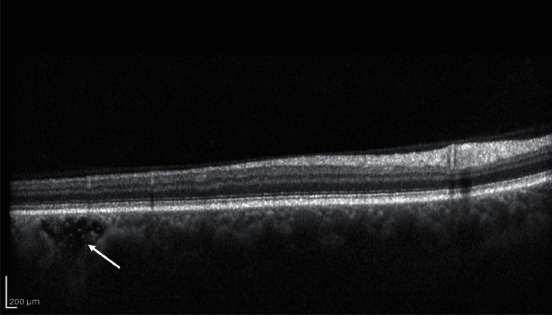
Structural biomarker: choroidal caverns (white arrow).

Choroidal caverns have been described with the following morphological features: (1) nonreflective spherical to polyhedral structures visible on en face and cross-sectional OCT; (2) posterior tail of hypertransmission on cross-sectional OCT (B-scan); (3) in case of RPE loss, frequently hyperreflective on Near Infrared Imaging and rarely reflective on color photographs or hyperfluorescent on ICGA; (4) not visible on FA or fundus autofluorescence imaging; (5) no evidence of flow signal on en face or cross-sectional OCT-A [109] (Figure 9).

A recent histological and clinical imaging study characterized and defined the morphology of these lesions. They could be present in healthy subjects, as well as in different degenerative retinal pathologies, characterized by retinal and RPE atrophy. [8] Choroidal caverns were first hypothesized to be nonperfused ghost vessels with preserved stromal pillars at sites of preexisting choroidal vessels [110]. Subsequent studies proposed them as OCT correlates of Friedman lipid-rich globules [111].

In AMD, these lesions have been described in association with geographic atrophy, as well as related to neovascular tissue. In this latter case, lipid globules can be found in the sub-RPE space, intramembrane, or now described in subretinal space, to be distinguished from subretinal fluid [112, 113].

Since sub-RPE caverns have not been associated with pathological significance, no clinical intervention is needed.

#### 3.2.4. Choroidal Thickness: Subfoveal Choroidal Thickness and Choroidal Volume

The evaluation of morphological parameters of the choroid has been enhanced with the latest Swept Source OCTs enabling the analysis beyond the RPE through a strong signal identifying the vascular layer boundaries [114]. The reproducibility of the measurement is still uncertain, probably due to the circadian variation of choroidal thickness, its sensibility to systemic pressure, and different ocular variables like pre- and post-anti-VEGF injection conditions [115].

A condition of choroidal hypoperfusion may be considered as an etiological factor for the development and progression of exudative AMD. This fact is still debated, considering the role of outer retina and RPE in choriocapillary vascular sclerosis [116].

**Figure 10:**
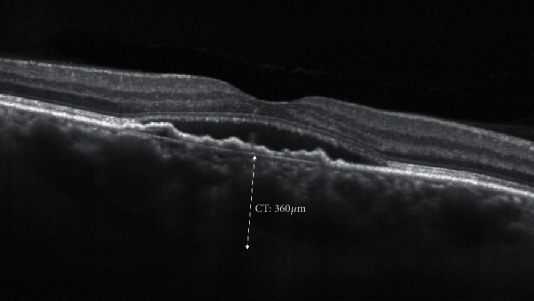
Structural biomarker: manual caliper to measure choroidal thickness (CT).

All choroidal parameters, in particular the widely studied subfoveal choroidal thickness (Figure 10), did not demonstrate a correlation with visual acuity recovery in treated patients, nor a different response to intravitreal anti-VEGF therapy. Choroidal volume, trying to avoid biases in choroidal boundary measurements, still does not show interesting clinical correlations [114, 117, 118].

A well-defined morphological entity has been identified in the pachychoroid condition. Pachychoroid is characterized by the presence of increased choroidal thickness associated with a dilation of the outer choroidal layer. Pachychoroid has been associated with a spectrum of clinical conditions, in particular central serous chorioretinopathy, and choroidal neovascularization (called pachychoroid neovasculopathy). These features differ from nAMD, even though polypoidal choroidal neovascularization may represent a common complication [119, 120].

#### 3.2.5. Choroidal Vascular Index (CVI)

Choroidal Vascular Index (CVI) has been elaborated with a binarization method using choroidal OCT B-scan to quantify the vascular component in the context of choroidal tissue overall. The imaging evaluation defined two components considering the tissue and the vascular lumen. The index is defined as the proportion of the lumen area over the total analyzed area of the scan. This parameter seems to be more stable than choroidal thickness measurements [121]. Indeed, considering the hypoxic hypothesis for AMD progression towards choroidal neovascularization, several studies correlated the reduction of CVI with the decrease of the vascular lumen area compared to the stromal area of the choroid [122, 123]. These preliminary data may have to be further verified comparing both eyes in the same patient or different cohorts of healthy and AMD patients, to enable a risk assessment for AMD development and progression.

## 4. Conclusions

OCT biomarkers are becoming even more useful in the management of patients affected by nAMD, for several reasons (Table 1).

Firstly, the identification of specific biomarkers at baseline foresees the visual prognosis of these patients, even before anti-VEGF treatment has started. Furthermore, biomarkers can provide information about the expected treatment response. In this regard, it is important to identify predictive factors associated with visual outcomes, since it could help the physician manage patients' expectations and make treatment decisions [124].

Secondly, biomarkers enable the evaluation of the progression of the disease and the treatment response, regardless of any VA alterations. In particular, the arrangement of retinal fluids can steer therapeutic decisions, with SRF being better tolerated than IRC. Indeed, OCT allows obtaining valuable information easily and quickly for an adequate course of treatment of nAMD patients, which cannot be ignored.

Moreover, OCT biomarkers can be useful to reduce the therapeutic burden of anti-VEGF treatments and to tailor the approach to each patient with nAMD.

Currently, hospital resources are not unlimited, even though there are a large number of patients needing anti-VEGF treatment. For this reason, resources must be allocated in the best possible way, ensuring an appropriate treatment to each patient [125]. Thus, for instance, it is worth prioritizing intravitreal treatment to those patients presenting biomarkers predicting good visual recovery.

Finally, biomarkers can help identify the right moment to stop intravitreal treatment for those patients having no further improvement. When OCT evaluation shows negative biomarkers, such as the disruption of the foveal photoreceptor layer, the presence of ORTs, and/or persistent IRC associated with persistent low visual acuity, physicians should stop anti-VEGF treatment, reallocating resources to other patients. On the contrary, when OCT shows positive biomarkers such as the disappearance of IRC, associated with persistent SRF or PED correlated to a good VA, the physician should continue anti-VEGF treatment.

In conclusion, OCT biomarkers are suitable to predict VA in patients with nAMD, and to guide the treatment and follow-up of these patients, improving the quality of nAMD management.

## Figures and Tables

**Table 1:** Summary of OCT biomarkers and prognostic value.

Biomarker		Role
Central retinal thickness		Limited prognostic value for visual acuity If increased correlation with lesion activity is higher
Distribution of retinal fluids	Intraretinal fluids (IRC)	Negative prognostic value for VA
	Subretinal fluids (SRF)	Role debated Protective for VA if chronic Related to a stable disease More treatments needed
	Sub-RPE fluids	No prognostic value for VA
Structural alterations	Subretinal hyperreflective material (SRHM)	Negative prognostic value, in particular if persistent after anti-VEGF therapy
	Outer retinal tubulation (ORT)	Associated to worse visual prognosis
	Photoreceptor layer damage	Negative prognosis on visual acuity
	Vitreomacular interface	VMA and VMT: more intravitreal treatments needed No prognostic value for visual acuity
	Hyperreflective dots	Related to activity of the lesion and its recurrences
	RPE rips	Negative prognostic value in particular if subfoveal
	Choroidal morphology	Choroidal thickness: no prognostic value on VA Prechoroidal clefts: risk for REP rips

## Data Availability

Research data will be available upon request from the corresponding author.

## References

[1] Branisteanu D., Branisteanu D., Feraru C. (2020). Influence of unilateral intravitreal bevacizumab injection on the incidence of symptomatic choroidal neovascularization in the fellow eye in patients with neovascular age‑related macular degeneration (review). *Experimental and Therapeutic Medicine*.

[2] Daien V., Finger R. P., Talks J. S. (2020). Evolution of treatment paradigms in neovascular age-related macular degeneration: a review of real-world evidence. *British Journal of Ophthalmology*.

[3] Stahl A. (2020). The diagnosis and treatment of age-related macular degeneration. *Deutsches Aerzteblatt Online*.

[4] Wong W. L., Su X., Li X. (2014). Global prevalence of age-related macular degeneration and disease burden projection for 2020 and 2040: a systematic review and meta-analysis. *The Lancet Global Health*.

[5] Singh S. R., Lupidi M., Mishra S. B., Paez-Escamilla M., Querques G., Chhablani J. (2020). Unique optical coherence tomographic features in age-related macular degeneration. *Survey of Ophthalmology*.

[6] Lai T.-T., Hsieh Y.-T., Yang C.-M., Ho T.-C., Yang C.-H. (2019). Biomarkers of optical coherence tomography in evaluating the treatment outcomes of neovascular age-related macular degeneration: a real-world study. *Scientific Reports*.

[7] Phadikar P., Saxena S., Ruia S., Lai T. Y. Y., Meyer C. H., Eliott D. (2017). The potential of spectral domain optical coherence tomography imaging based retinal biomarkers. *International Journal of Retina and Vitreous*.

[8] Schmidt-Erfurth U., Waldstein S. M. (2016). A paradigm shift in imaging biomarkers in neovascular age-related macular degeneration. *Progress in Retinal and Eye Research*.

[9] Chakravarthy U., Havilio M., Syntosi A. (2021). Impact of macular fluid volume fluctuations on visual acuity during anti-VEGF therapy in eyes with nAMD. *Eye*.

[10] Ho A. C., Saroj N., Baker K. (2018). Impact of baseline characteristics on treatment response to intravitreal aflibercept injection for wet age-related macular degeneration. *Ophthalmology Retina*.

[11] Ou W. C., Brown D. M., Payne J. F., Wykoff C. C. (2017). Relationship between visual acuity and retinal thickness during anti-vascular endothelial growth factor therapy for retinal diseases. *American Journal of Ophthalmology*.

[12] Moraes G., Fu D. J., Wilson M. (2021). Quantitative analysis of OCT for neovascular age-related macular degeneration using deep learning. *Ophthalmology*.

[13] Jaffe G. J., Martin D. F., Toth C. A. (2013). Macular morphology and visual acuity in the comparison of age-related macular degeneration treatments trials. *Ophthalmology*.

[14] Sharma S., Toth C. A., Daniel E. (2016). Macular morphology and visual acuity in the second year of the comparison of age-related macular degeneration treatments trials. *Ophthalmology*.

[15] Ritter M., Simader C., Bolz M. (2014). Intraretinal cysts are the most relevant prognostic biomarker in neovascular age-related macular degeneration independent of the therapeutic strategy. *British Journal of Ophthalmology*.

[16] Sadda S. R., Tuomi L. L., Ding B., Fung A. E., Hopkins J. J. (2018). Macular atrophy in the HARBOR study for neovascular age-related macular degeneration. *Ophthalmology*.

[17] Spaide R. F., Jaffe G. J., Sarraf D. (2020). Consensus nomenclature for reporting neovascular age-related macular degeneration data: consensus on neovascular age-related macular degeneration nomenclature study group. *Ophthalmology*.

[18] Waldstein S. M., Simader C., Staurenghi G. (2016). Morphology and visual acuity in aflibercept and ranibizumab therapy for neovascular age-related macular degeneration in the VIEW trials. *Ophthalmology*.

[19] Schmidt-Erfurth U., Waldstein S. M., Deak G.-G., Kundi M., Simader C. (2015). Pigment epithelial detachment followed by retinal cystoid degeneration leads to vision loss in treatment of neovascular age-related macular degeneration. *Ophthalmology*.

[20] Schmidt-Erfurth U., Klimscha S., Waldstein S. M., Bogunović H. (2017). A view of the current and future role of optical coherence tomography in the management of age-related macular degeneration. *Eye*.

[21] Hosseini H., Rabina G., Pettenkofer M. (2020). Clinical characteristics and visual outcomes of non-resolving subretinal fluid in neovascular AMD despite continuous monthly anti-VEGF injections: a long-term follow-up. *Graefe’s Archive for Clinical and Experimental Ophthalmology*.

[22] Gianniou C., Dirani A., Jang L., Mantel I. (2015). Refractory intraretinal or subretinal fluid in neovascular age-related macular degeneration treated with intravitreal ranizubimab: functional and structural outcome. *Retina*.

[23] Arnold J. J., Markey C. M., Kurstjens N. P., Guymer R. H. (2016). The role of sub-retinal fluid in determining treatment outcomes in patients with neovascular age-related macular degeneration—a phase IV randomised clinical trial with ranibizumab: the FLUID study. *BMC Ophthalmology*.

[24] Guymer R. H., Markey C. M., McAllister I. L., Gillies M. C., Hunyor A. P., Arnold J. J. (2019). Tolerating subretinal fluid in neovascular age-related macular degeneration treated with ranibizumab using a treat-and-extend regimen: FLUID study 24-month results. *Ophthalmology*.

[25] Roh M., Laíns I., Shin H. J. (2019). Microperimetry in age-related macular degeneration: association with macular morphology assessed by optical coherence tomography. *British Journal of Ophthalmology*.

[26] Sadda S. R., Guymer R., Monés J. M., Tufail A., Jaffe G. J. (2020). Anti–vascular endothelial growth factor use and atrophy in neovascular age-related macular degeneration: systematic literature review and expert opinion. *Ophthalmology*.

[27] Jaffe G. J., Ying G.-S., Toth C. A. (2019). Macular morphology and visual acuity in year five of the comparison of age-related macular degeneration treatments trials. *Ophthalmology*.

[28] Cheong K. X., Grewal D. S., Teo K. Y. C., Gan A. T. L., Jaffe G. J., Cheung G. C. M. (2020). The relationship between pigment epithelial detachment and visual outcome in neovascular age-related macular degeneration and polypoidal choroidal vasculopathy. *Eye*.

[29] Simader C., Ritter M., Bolz M. (2014). Morphologic parameters relevant for visual outcome during anti-angiogenic therapy of neovascular age-related macular degeneration. *Ophthalmology*.

[30] Ashraf M., Souka A., Adelman R. A. (2018). Age-related macular degeneration: using morphological predictors to modify current treatment protocols. *Acta Ophthalmologica*.

[31] Sarraf D., London N. J. S., Khurana R. N. (2016). Ranibizumab treatment for pigment epithelial detachment secondary to neovascular age-related macular degeneration: post hoc analysis of the HARBOR study. *Ophthalmology*.

[32] Karampelas M., Malamos P., Petrou P., Georgalas I., Papaconstantinou D., Brouzas D. (2020). Retinal pigment epithelial detachment in age-related macular degeneration. *Ophthalmology and Therapy*.

[33] Ach T., Hoeh A. E., Ruppenstein M., Kretz F. T. A., Dithmar S. (2010). Intravitreal bevacizumab in vascular pigment epithelium detachment as a result of subfoveal occult choroidal neovascularization in age-related macular degeneration. *Retina*.

[34] Curcio C. A., Medeiros N. E., Millican C. L. (1996). Photoreceptor loss in age-related macular degeneration. *Investigative Ophthalmology & Visual Science*.

[35] Otani T., Yamaguchi Y., Kishi S. (2010). Correlation between visual acuity and foveal microstructural changes in diabetic macular edema. *Retina*.

[36] Shin H. J., Lee S. H., Chung H., Kim H. C. (2012). Association between photoreceptor integrity and visual outcome in diabetic macular edema. *Graefe’s Archive for Clinical and Experimental Ophthalmology*.

[37] Chhablani J. K., Kim J. S., Cheng L., Kozak I., Freeman W. (2012). External limiting membrane as a predictor of visual improvement in diabetic macular edema after pars plana vitrectomy. *Graefe’s Archive for Clinical and Experimental Ophthalmology*.

[38] Oishi A., Tsujikawa A., Yamashiro K. (2015). One-year result of aflibercept treatment on age-related macular degeneration and predictive factors for visual outcome. *American Journal of Ophthalmology*.

[39] Oishi A., Shimozono M., Mandai M., Hata M., Nishida A., Kurimoto Y. (2013). Recovery of photoreceptor outer segments after anti-VEGF therapy for age-related macular degeneration. *Graefe’s Archive for Clinical and Experimental Ophthalmology*.

[40] Itoh Y., Inoue M., Rii T., Hirota K., Hirakata A. (2013). Correlation between foveal cone outer segment tips line and visual recovery after epiretinal membrane surgery. *Investigative Opthalmology & Visual Science*.

[41] Woronkowicz M., Lightman S., Tomkins-Netzer O. (2020). The prognostic value of total macular external limiting membrane and ellipsoid zone damage for clinical outcome in treatment-resistant neovascular age-related macular degeneration. *Graefe’s Archive for Clinical and Experimental Ophthalmology*.

[42] Coscas F., Coscas G., Lupidi M. (2015). Restoration of outer retinal layers after aflibercept therapy in exudative AMD: prognostic value. *Investigative Opthalmology & Visual Science*.

[43] Shin H. J., Chung H., Kim H. C. (2011). Association between foveal microstructure and visual outcome in age-related macular degeneration. *Retina*.

[44] Riedl S., Cooney L., Grechenig C. (2020). Topographic analysis of photoreceptor loss correlated with disease morphology in neovascular age-related macular degeneration. *Retina*.

[45] Kim Y. M., Kim J. H., Koh H. J. (2012). Improvement of photoreceptor integrity and associated visual outcome in neovascular age-related macular degeneration. *American Journal of Ophthalmology*.

[46] Hwang H. S., Chae J. B., Kim J. Y., Kim D. Y. (2017). Association between hyperreflective dots on spectral-domain optical coherence tomography in macular edema and response to treatment. *Investigative Opthalmology & Visual Science*.

[47] Coscas G., Coscas F., Vismara S., Zourdani A., Li Calzi C. (2009). Clinical features and natural history of AMD on OCT. *Optical Coherence Tomography in Age-Related Macular Degeneration*.

[48] Curcio C. A., Zanzottera E. C., Ach T., Balaratnasingam C., Freund K. B. (2017). Activated retinal pigment epithelium, an optical coherence tomography biomarker for progression in age-related macular degeneration. *Investigative Ophthalmology & Visual Science*.

[49] Balaratnasingam C., Messinger J. D., Sloan K. R., Yannuzzi L. A., Freund K. B., Curcio C. A. (2017). Histologic and optical coherence tomographic correlates in drusenoid pigment epithelium detachment in age-related macular degeneration. *Ophthalmology*.

[50] Altay L., Scholz P., Schick T. (2016). Association of hyperreflective foci present in early forms of age-related macular degeneration with known age-related macular degeneration risk polymorphisms. *Investigative Opthalmology & Visual Science*.

[51] Coscas G., De Benedetto U., Coscas F. (2013). Hyperreflective dots: a new spectral-domain optical coherence tomography entity for follow-up and prognosis in exudative age-related macular degeneration. *Ophthalmologica*.

[52] Zweifel S. A., Engelbert M., Laud K., Margolis R., Spaide R. F., Freund K. B. (2009). Outer retinal tubulation: a novel optical coherence tomography finding. *Archives of Ophthalmology*.

[53] Lee J. Y., Folgar F. A., Maguire M. G. (2014). Outer retinal tubulation in the comparison of age-related macular degeneration treatments trials (CATT). *Ophthalmology*.

[54] Dirani A., Gianniou C., Marchionno L., Decugis D., Mantel I. (2015). Incidence of outer retinal tubulation in ranibizumab-treated age-related macular degeneration. *Retina*.

[55] Litts K. M., Messinger J. D., Dellatorre K., Yannuzzi L. A., Freund K. B., Curcio C. A. (2015). Clinicopathological correlation of outer retinal tubulation in age-related macular degeneration. *JAMA Ophthalmology*.

[56] Schaal K. B., Freund K. B., Litts K. M., Zhang Y., Messinger J. D., Curcio C. A. (2015). Outer retinal tubulation in advanced age-related macular degeneration: optical coherence tomographic findings correspond to histology. *Retina*.

[57] Preti R. C., Govetto A., Filho R. G. A. (2018). Optical coherence tomography analysis of outer retinal tubulations: sequential evolution and pathophysiological insights. *Retina*.

[58] Kovacs A., Kiss T., Rarosi F., Somfai G. M., Facsko A., Degi R. (2018). The effect of ranibizumab and aflibercept treatment on the prevalence of outer retinal tubulation and its influence on retreatment in neovascular age-related macular degeneration. *BMC Ophthalmology*.

[59] Willoughby A. S., Ying G. S., Toth C. A. (2015). Subretinal hyperreflective material in the comparison of age-related macular degeneration treatments trials. *Ophthalmology*.

[60] Ying G.-s., Kim B. J., Maguire M. G. (2014). Sustained visual acuity loss in the comparison of age-related macular degeneration treatments trials. *JAMA Ophthalmology*.

[61] Ristau T., Keane P. A., Walsh A. C. (2014). Relationship between visual acuity and spectral domain optical coherence tomography retinal parameters in neovascular age-related macular degeneration. *Ophthalmologica*.

[62] Charafeddin W., Nittala M. G., Oregon A., Sadda S. R. (2015). Relationship between subretinal hyperreflective material reflectivity and volume in patients with neovascular age-related macular degeneration following anti-vascular endothelial growth factor treatment. *Ophthalmic Surgery, Lasers and Imaging Retina*.

[63] Pokroy R., Mimouni M., Barayev E. (2018). Prognostic value of subretinal hyperreflective material in neovascular age-related macular degeneration treated with bevacizumab. *Retina*.

[64] Dansingani K. K., Tan A. C. S., Gilani F. (2016). Subretinal hyperreflective material imaged with optical coherence tomography angiography. *American Journal of Ophthalmology*.

[65] Kawashima Y., Hata M., Oishi A. (2017). Association of vascular versus avascular subretinal hyperreflective material with aflibercept response in age-related macular degeneration. *American Journal of Ophthalmology*.

[66] Kumar J. B., Stinnett S., Han J. I. L., Jaffe G. J. (2020). Correlation of subretinal hyperreflective material morphology and visual acuity in neovascular age-related macular degeneration. *Retina*.

[67] Invernizzi A., Nguyen V., Arnold J., Young S., Barthelmes D., Gillies M. C. (2018). Early and late retinal pigment epithelium tears after anti-vascular endothelial growth factor therapy for neovascular age-related macular degeneration. *Ophthalmology*.

[68] Sastre-Ibáñez M., Martínez-Rubio C., Molina-Pallete R. (2019). Retinal pigment epithelial tears. *Journal Français d’Ophtalmologie*.

[69] Cho H. J., Kim H. S., Yoo S. G. (2016). Retinal pigment epithelial tear after intravitreal ranibizumab treatment for neovascular age-related macular degeneration. *Retina*.

[70] Chan C. K., Abraham P., Meyer C. H. (2010). Optical coherence tomography-measured pigment epithelial detachment height as a predictor for retinal pigment epithelial tears associated with intravitreal bevacizumab injections. *Retina*.

[71] Sarraf D., Chan C., Rahimy E., Abraham P. (2013). Prospective evaluation of the incidence and risk factors for the development of RPE tears after high- and low-dose ranibizumab therapy. *Retina*.

[72] Chiang A., Chang L. K., Yu F., Sarraf D. (2008). Predictors of anti-VEGF-associated retinal pigment epithelial tear using FA and OCT analysis. *Retina*.

[73] Chan C. K., Meyer C. H., Gross J. G. (2007). Retinal pigment epithelial tears after intravitreal bevacizumab injection for neovascular age-related macular degeneration. *Retina*.

[74] Moroz I., Moisseiev J., Alhalel A. (2009). Optical coherence tomography predictors of retinal pigment epithelial tear following intravitreal bevacizumab injection. *Ophthalmic Surgery, Lasers and Imaging Retina*.

[75] Shiraki K., Kohno T., Ataka S., Abe K., Inoue K., Miki T. (2001). Thinning and small holes at an impending tear of a retinal pigment epithelial detachment. *Graefe’s Archive for Clinical and Experimental Ophthalmology*.

[76] Musashi K., Tsujikawa A., Hirami Y. (2007). Microrips of the retinal pigment epithelium in polypoidal choroidal vasculopathy. *American Journal of Ophthalmology*.

[77] Ersoz M. G., Karacorlu M., Arf S., Sayman Muslubas I., Hocaoglu M. (2017). Retinal pigment epithelium tears: classification, pathogenesis, predictors, and management. *Survey of Ophthalmology*.

[78] Rachitskaya A. V., Goldhardt R. (2015). Retinal pigment epithelium tear. *Current Ophthalmology Reports*.

[79] Nicolò M., Ghiglione D., Calabria G. (2006). Retinal pigment epithelial tear following intravitreal injection of bevacizumab (avastin). *European Journal of Ophthalmology*.

[80] Querques G., Capuano V., Costanzo E. (2016). Retinal pigment epithelium aperture: a previously unreported finding in the evolution of avascular pigment epithelium detachment. *Retina*.

[81] Mukai R., Sato T., Kishi S. (2015). Repair mechanism of retinal pigment epithelial tears in age-related macular degeneration. *Retina*.

[82] Oishi A., Fang P. P., Thiele S., Holz F. G., Krohne T. U. (2018). Longitudinal change of outer nuclear layer after retinal pigment epithelial tear secondary to age-related macular degeneration. *Retina*.

[83] Vazquez-Alfageme C., Nicholson L., Hamilton R. D., Patel P. J. (2019). Incidence and long-term visual acuity outcomes of retinal pigment epithelium tears after intravitreal anti-vascular endothelial growth factor treatment of neovascular age-related macular degeneration. *Retina*.

[84] Maier M., Pfrommer S., Burzer S., Feucht N., Winkler von Mohrenfels C., Lohmann C. (2012). Vitreomacular interface and posterior vitreomacular adhesion in exudative age-related macular degeneration (AMD): an OCT-based comparative study. *Klinische Monatsblätter für Augenheilkunde*.

[85] Ashraf M., Souka A., Adelman R. A. (2017). Association between the vitreomacular interface and optical coherence tomography characteristics in wet age-related macular degeneration. *Retina*.

[86] Gattoussi S., Buitendijk G. H. S., Peto T. (2019). The European eye epidemiology spectral-domain optical coherence tomography classification of macular diseases for epidemiological studies. *Acta Ophthalmologica*.

[87] Maggio E., Polito A., Guerriero M., Prigione G., Parolini B., Pertile G. (2017). Vitreomacular adhesion and the risk of neovascular age-related macular degeneration. *Ophthalmology*.

[88] Kang E. C., Koh H. J. (2015). Effects of vitreomacular adhesion on age-related macular degeneration. *Journal of Ophthalmology*.

[89] Kanadani T. C. M., Dos Reis Veloso C. E., Dorairaj S., Nehemy M. B. (2017). Influence of vitreomacular adhesion on anti-vascular endothelial growth factor treatment for neovascular age-related macular degeneration. *Ophthalmic Research*.

[90] Gattoussi S., Cougnard-Grégoire A., Delyfer M.-N. (2017). Vitreomacular adhesion and its association with age-related macular degeneration in a population-based setting: the alienor study. *Investigative Opthalmology & Visual Science*.

[91] Jackson T. L., Nicod E., Angelis A. (2013). Vitreous attachment in age-related macular degeneration, diabetic macular edema, and retinal vein occlusion: a systematic review and metaanalysis. *Retina*.

[92] El-Hifnawy M. A., Ibrahim H. A., Gomaa A. R., Elmasry M. A. (2017). The vitreomacular interface in different types of age-related macular degeneration. *International Journal of Ophthalmology*.

[93] Jun Lee S., Lee C. S., Jun Koh H. (2009). Posterior vitreomacular adhesion and risk of exudative age-related macular degeneration: paired eye study. *American Journal of Ophthalmology*.

[94] Novack R. L., Staurenghi G., Girach A., Narendran N., Tolentino M. (2015). Safety of intravitreal ocriplasmin for focal vitreomacular adhesion in patients with exudative age-related macular degeneration. *Ophthalmology*.

[95] Lee S. J., Koh H. J. (2011). Effects of vitreomacular adhesion on anti-vascular endothelial growth factor treatment for exudative age-related macular degeneration. *Ophthalmology*.

[96] Gao M., Liu L., Liang X., Yu Y., Liu X., Liu W. (2017). Influence of vitreomacular interface on anti-vascular endothelial growth factor treatment outcomes in neovascular age-related macular degeneration: a MOOSE-compliant meta-analysis. *Medicine*.

[97] Xie P., Zheng X., Yu Y. (2017). Vitreomacular adhesion or vitreomacular traction may affect antivascular endothelium growth factor treatment for neovascular age-related macular degeneration. *British Journal of Ophthalmology*.

[98] Green-Simms A. E., Bakri S. J. (2011). Vitreomacular traction and age-related macular degeneration. *Seminars in Ophthalmology*.

[99] Kimura S., Morizane Y., Toshima S. (2016). Efficacy of vitrectomy and inner limiting membrane peeling in age-related macular degeneration resistant to anti-vascular endothelial growth factor therapy, with vitreomacular traction or epiretinal membrane. *Graefe’s Archive for Clinical and Experimental Ophthalmology*.

[100] Lee K. H., Chin H. S., Kim N. R., Moon Y. S. (2015). Effects of vitreomacular traction on ranibizumab treatment response in eyes with neovascular age-related macular degeneration. *Korean Journal of Ophthalmology*.

[101] Krishnan R., Arora R., De Salvo G. (2015). Vitreomacular traction affects anti-vascular endothelial growth factor treatment outcomes for exudative age-related macular degeneration. *Retina*.

[102] Padnick-Silver L., Weinberg A. B., Lafranco F. P., Macsai M. S. (2012). Pilot study for the detection of early exudative age-related macular degeneration with optical coherence tomography. *Retina*.

[103] Silva R., Cachulo M. L., Fonseca P. (2011). Age-related macular degeneration and risk factors for the development of choroidal neovascularisation in the fellow eye: a 3-year follow-up study. *Ophthalmologica*.

[104] Rahimy E., Freund K. B., Larsen M. (2014). Multilayered pigment epithelial detachment in neovascular age-related macular degeneration. *Retina*.

[105] Kim J. M., Kang S. W., Son D. y., Bae K. (2017). Risk factors and clinical significance of prechoroidal cleft in neovascular age-related macular degeneration. *Retina*.

[106] Nagiel A., Freund K. B., Spaide R. F., Munch I. C., Larsen M., Sarraf D. (2013). Mechanism of retinal pigment epithelium tear formation following intravitreal anti-vascular endothelial growth factor therapy revealed by spectral-domain optical coherence tomography. *American Journal of Ophthalmology*.

[107] Kim J. H., Chang Y. S., Kim J. W., Kim C. G., Lee D. W. (2018). Prechoroidal cleft in type 3 neovascularization: incidence, timing, and its association with visual outcome. *Journal of Ophthalmology*.

[108] Khan S., Engelbert M., Imamura Y., Freund K. B. (2012). Polypoidal choroidal vasculopathy: simultaneous indocyanine green angiography and eye-tracked spectral domain optical coherence tomography findings. *Retina*.

[109] Dolz-Marco R., Glover J. P., Gal-Or O. (2018). Choroidal and sub-retinal pigment epithelium caverns: multimodal imaging and correspondence with friedman lipid globules. *Ophthalmology*.

[110] Querques G., Costanzo E., Miere A., Capuano V., Souied E. H. (2016). Choroidal caverns: a novel optical coherence tomography finding in geographic atrophy. *Investigative Opthalmology & Visual Science*.

[111] Friedman E., Smith T. R. (1966). Clinical and pathological study of choroidal lipid globules. *Archives of Ophthalmology*.

[112] Xu X., Liu X., Wang X. (2017). Retinal pigment epithelium degeneration associated with subretinal drusenoid deposits in age-related macular degeneration. *American Journal of Ophthalmology*.

[113] Fernández-Avellaneda P., Freund K. B., Wang R. K. (2021). Multimodal imaging features and clinical relevance of subretinal lipid globules. *American Journal of Ophthalmology*.

[114] Wang J., Yin L. R. (2020). The application of enhanced depth imaging spectral-domain optical coherence tomography in macular diseases. *Journal of Ophthalmology*.

[115] Singh S. R., Vupparaboina K. K., Goud A., Dansingani K. K., Chhablani J. (2019). Choroidal imaging biomarkers. *Survey of Ophthalmology*.

[116] Wang X., Zeng L., Chen M., Liu L. (2020). Choroidal vascular changes in age-related macular degeneration: a protocol for systematic review and meta-analysis. *Medicine*.

[117] Kanadani T. C. M., Veloso C. E., Nehemy M. B. (2018). Subfoveal choroidal thickness in eyes with neovascular age-related macular degeneration treated with anti-vascular endothelial growth factor agents. *Ophthalmologica*.

[118] Koizumi H., Kano M., Yamamoto A. (2016). Subfoveal choroidal thickness during aflibercept therapy for neovascular age-related macular degeneration: twelve-month results. *Ophthalmology*.

[119] Cheung C. M. G., Lee W. K., Koizumi H., Dansingani K., Lai T. Y. Y., Freund K. B. (2019). Pachychoroid disease. *Eye*.

[120] Pang C. E., Freund K. B. (2015). Pachychoroid neovasculopathy. *Retina*.

[121] Agrawal R., Gupta P., Tan K.-A., Cheung C. M. G., Wong T.-Y., Cheng C.-Y. (2016). Choroidal vascularity index as a measure of vascular status of the choroid: measurements in healthy eyes from a population-based study. *Scientific Reports*.

[122] Wei X., Ting D. S. W., Ng W. Y., Khandelwal N., Agrawal R., Cheung C. M. G. (2017). Choroidal vascularity index: a novel optical coherence tomography based parameter in patients with exudative age-related macular degeneration. *Retina*.

[123] Gupta P., Ting D. S. W., Thakku S. G. (2017). Detailed characterization of choroidal morphologic and vascular features in age-related macular degeneration and polypoidal choroidal vasculopathy. *Retina*.

[124] Midena E., Varano M., Pilotto E. (2021). Real-life patient journey in neovascular age-related macular degeneration: a narrative medicine analysis in the Italian setting. *Eye*.

[125] Li E., Donati S., Lindsley K. B., Krzystolik M. G., Virgili G. (2020). Treatment regimens for administration of anti-vascular endothelial growth factor agents for neovascular age-related macular degeneration. *The Cochrane Database of Systematic Reviews*.

